# Non-Invasive High-Resolution Imaging of In Vivo Human Myelinated Axons

**DOI:** 10.3390/diagnostics14030253

**Published:** 2024-01-24

**Authors:** Marco Lombardo, Massimo Cesareo, Benedetto Falsini, Andrea Cusumano

**Affiliations:** 1 Department of Experimental Medicine, Ophthalmology Unit, University of Rome Tor Vergata, 00133 Rome, Italy; massimo.cesareo@uniroma2.it (M.C.); cusumano@cusumano.com (A.C.); 2 Macula & Genoma Foundation, 00133 Rome, Italy; bfalsini@gmail.com; 3 Macula & Genoma Foundation USA, New York, NY 10017, USA

**Keywords:** adaptive optics, myelinated retinal nerve fiber, myelin sheath, ocular imaging

## Abstract

This work aims to reveal the microscopic (2–3 micrometer resolution) appearance of human myelinated nerve fibers in vivo for the first time. We analyzed the myelinated retinal nerve fibers of a male patient without other neurological disorders in a non-invasive way using the transscleral optical phase imaging method with adaptive optics. We also analyzed the fellow eye with non-myelinated nerve fibers and compared the results with traditional ocular imaging methods such as optical coherence tomography. We documented the microscopic appearance of human myelin and myelinated axons in vivo. This method allowed us to obtain better details than through traditional ocular imaging methods. We hope these findings will be useful to the scientific community to evaluate neuro-retinal structures through new imaging techniques and more accurately document nerve anatomy and the pathophysiology of this disease.

**Figure 1:**
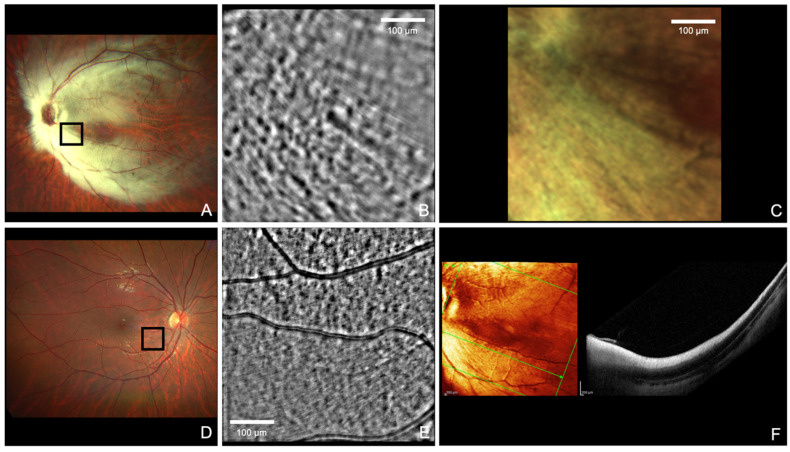
In vivo non-invasive image of myelinated nerve fibers at the emergence from the optic nerve of a human patient affected by myelinated retinal nerve fiber layer (MRNFL) acquired through the transscleral optical phase imaging (TOPI) method [1,2]. The myelination of retinal ganglion cell fibers normally proceeds from the lateral geniculate nucleus anteriorly to the lamina cribrosa of the eye. MRNFL occurs when, for reasons still unclear, this process extends beyond this limit and myelinated axons are visible on fundus examination [3]. Panel (**A**) shows the fundus retinography acquired with Clarus 500 (Carl Zeiss, Dublin, CA, USA) of the left eye affected by MRNFL. Panel (**B**) shows the image’s detail of the black square of panel (**A**) acquired by Cellularis Discovery (EarlySight SA, Geneva, Switzerland) using the TOPI method. It shows axon bundles aligned in the direction of the optic nerve that are larger probably because they are surrounded by the myelin sheath; myelinated nerve fibers had never previously been documented in a living human subject. Panel (**C**) shows the same detail as panel (**B**), acquired through the MultiColor acquisition of an optical coherence tomography (OCT) device (Spectralis HRA + OCT; Heidelberg Engineering). In comparison, the image resolution appears significantly lower than in panel B. This difference could be due to the patient’s high refractive error (−20 diopters), from which the resolution of the TOPI technology appears to be less affected, rather than to a better resolution of the technology. Panel (**D**) shows the fundus retinography of the fellow healthy eye acquired by Clarus 500. Panel (**E**) shows the image’s detail of the black square of panel (**D**) acquired with TOPI adaptive optics: the axons are normally non-myelinated and therefore smaller compared to the affected eye. Panel (**F**) shows an OCT B-scan passing through the affected area: the high reflectivity of MRNFL does not allow for a good resolution of the myelinated fibers. The patient, a 15-year-old Caucasian male, was in good health with no apparent major neurological findings. The right eye was within normal limits; the left eye was affected by MRNFL, myopia, and amblyopia (Straatsma syndrome [4,5]). Electrophysiological tests conducted according to the International Society for Clinical Electrophysiology of Vision (ISCEV) protocol showed reduced pattern electroretinogram (PERG) amplitudes and increased latencies in the affected eye compared to normal values. The most affected parameter was PERG amplitude, suggesting that the myelin sheath may interfere with the function of retinal ganglion cells. The broader significance of this report is to have documented for the first time the microscopic appearance (2–3 micrometer resolution) of myelinated axons in vivo. This method, at least in Straatsma syndrome, allows the examiner to evaluate in much greater detail, compared to conventional methods, the size and density of the myelinated fibers, potentially predicting their impact on inner retinal function and, more in general, on visual function. Although we considered it clinically relevant to analyze for the first time the appearance of MRNFL with adaptive optics technology, en-face OCT imaging can conceivably provide a similar resolution, especially when 3 × 3 mm OCTA patterns with image averaging are used. We hope these results will be useful to the scientific community for evaluating neuro-retinal structures through new imaging techniques and more accurately document nerve anatomy and the pathophysiology of this disease.

## Data Availability

The datasets generated during the current work are available from the corresponding author upon reasonable request.
